# Inherent variation of functional traits in winter and summer leaves of Mediterranean seasonal dimorphic species: evidence of a ‘within leaf cohort’ spectrum

**DOI:** 10.1093/aobpla/ply027

**Published:** 2018-04-24

**Authors:** Giacomo Puglielli, Laura Varone

**Affiliations:** Department of Environmental Biology, Sapienza University of Rome, Rome, Italy

**Keywords:** *Cistus*, deciduous, evergreen, leaf cohorts, leaf economic spectrum, LMA, leaf nitrogen, leaf payback time

## Abstract

The covariation pattern among leaf functional traits involved in resource acquisition has been successfully provided by the leaf economic spectrum (LES). Nevertheless, some aspects such as how the leaf trait variation sources affect LES predictions are still little investigated. Accordingly, the aim of this paper was to test whether leaf trait variations within different leaf cohorts could alter LES. Improving this knowledge can extend the potential of trait-based approaches in simulating future climate effects on ecosystems. A database on leaf morphological and physiological traits from different leaf cohorts of *Cistus* spp. was built by collecting data from literature. These species are seasonal dimorphic shrubs with two well-defined leaf cohorts during a year: summer leaves (SL) and winter leaves (WL). Traits included: leaf mass area (LMA), leaf thickness (LT), leaf tissue density (LTD), net photosynthetic rate on area (A_a_) and mass (A_m_) base, nitrogen content on area (N_a_) and mass (N_m_) base. The obtained patterns were analysed by standardized major axis regression and then compared with the global spectrum of evergreens and deciduous species. Climatic variable effect on leaf traits was also tested. Winter leaves and SL showed a great inherent variability for all the considered traits. Nevertheless, some relationships differed in terms of slopes or intercepts between SL and WL and between leaf cohorts and the global spectrum of evergreens and deciduous. Moreover, climatic variables differently affected leaf traits in SL and WL. The results show the existence of a ‘within leaf cohort’ spectrum, providing the first evidence on the role of leaf cohorts as LES source of variation. In fact, WL showed a high return strategy as they tended to maximize, in a short time, resource acquisition with a lower dry mass investment, while SL were characterized by a low return strategy.

## Teaser

Leaf morphological, physiological and biochemical traits tend to vary in a coordinated manner as described by the leaf economics spectrum (LES). However, LES is scale-dependent. In this paper we demonstrate that different leaf cohorts of evergreen seasonal dimorphic species display significant differences from the LES. Moreover, we show that species characterized by an extended leaf production during a growing season, such as evergreens, can shift from a more competitive to a more stress-tolerant strategy during a single growing season. This provides a new framework to better investigate evergreens’ future responses to the major drivers of global climate change.

## Introduction

Plant functional diversity is achieved through a suite of physiological and morphological traits, which contribute to define plant adaptive strategies to cope with environmental variations, and therefore to allow plant survival. Many of these traits are considered ‘economic traits’ being related to the capacity to acquire, use and conserve resources (Reich *et al.* 2003; Reich 2014). In the last two decades, a goal of plant ecologists has been to identify the trade-offs between functional traits in order to elucidate how the coordination among them could drive the plant response to environmental changes. In particular, the attention has been addressed to ‘leaf traits’ that have a key role in the carbon fixation strategy (Grime *et al.* 1997; Reich *et al.* 1997; Westoby *et al.* 2002; Ackerly 2004).

In 2004, the paper from Wright *et al.* provided a breakthrough in explaining the pattern of leaf trait covariation. The authors, by analysing six key leaf traits (leaf mass per area, maximum photosynthetic rate, leaf nitrogen content, leaf phosphorus content, dark respiration rate and leaf lifespan), suggested the existence of a spectrum of trade-offs between physiological, chemical and structural leaf traits (i.e. leaf economic spectrum, LES). Briefly, Wright *et al.* (2004) showed that the investments of plants in structural and chemical leaf traits have a return in terms of physiological activity. The spectrum goes from plant species that have a high return in physiological activity (i.e. high leaf nutrient content and high photosynthetic rate) to species with a lower potential rate of return (i.e. low leaf nutrient content, low photosynthetic rate). In terms of leaf morphology, the high return rate is related to less tough leaves (i.e. low leaf mass area, leaf thickness (LT) and leaf tissue density (LTD)) while the opposite is true for leaves characterized by a low return strategy. The strong point of LES was that it has been built on a large database (i.e. Glopnet), composed by 2548 species from 219 families and from 175 sites, covering biomes from the artic to the tropics. Given the size of the Glopnet, it has been possible to show that LES works globally independently of growth form, plant functional types or biome (Wright *et al.* 2004). Thus, LES is worthy to describe plants strategies through the observed leaf functional trait relationships, providing insights to explain species growth and survival across resource availability gradients (Reich 2014).

Despite the LES effectiveness, some aspects are still little investigated (Blonder *et al.* 2013; Reich 2014; Niinemets 2015; Keenan and Niinemets 2016), especially those concerning the leaf trait variation sources. In its original description, the pattern of leaf interspecific variation along the spectrum is essentially consistent with the characteristics of the sites where species were sampled (Niinemets 2015). However, when studies are carried out at a different scale, such as within-species variation, the covariation patterns may not agree with LES predictions (Niinemets 2015; Keenan and Niinemets 2016). For example, Wright and Sutton-Grier (2012) found that the LES relationships were weak in local communities exposed to environmental changes. Similarly, Keenan and Niinemets (2016) by using a worldwide database of within-canopy plasticity showed that in response to a light gradient some relationships such as those between photosynthetic rates per area (A_a_) and leaf mass per area did not follow the LES. Yet, another not well-investigated aspect is whether leaf traits within individuals vary according to LES (Blonder *et al.* 2013). Understanding the LES robustness at different scales within individuals could help to improve ecological predictions on plant community responses to environmental changes because it would highlight the underlying physiological causes of the trait correlations. Also, expanding the knowledge on the sources of variation of the LES would make it possible to widen the potential of the trait-based approaches in simulating future climate effects on ecosystems (Scheiter and Higgins 2009; Niinemets *et al.* 2011; Niinemets 2015).

Evergreen species are characterized by differences in leaf traits depending on flushes (i.e. leaf cohorts, Morales *et al.* 2014) and Niinemets (2014) argued that the timing of leaf flush may be an important driver altering the LES in evergreens. Identifying the sources of leaf variation is particularly interesting for Mediterranean evergreen species, which face strong seasonal climatic fluctuations. However, whether leaf cohorts can be considered as a source of variation of the LES has not yet been tested. This gap of knowledge mainly arises because of the difficulty to collect data on different leaf cohorts in a wide range of species and environmental conditions.

Starting from these last considerations, the aim of our paper is to fill this gap of knowledge by investigating the effectiveness of LES within leaf cohorts in *Cistus* spp. as well as to understand the climatic control on their leaf cohort traits. Many characteristics of *Cistus* spp. make them key components of the Mediterranean ecosystems. They developed with the advent of the Mediterranean climate and were determinants of the composition and current diversity of the Mediterranean area (Gratani and Varone 2004; Correia and Ascensão 2017). Indeed, they are pioneer species with a high germination rate and seedling recruitment after fires (de Dato *et al.* 2013), acting as a source of nutrients to the soil and facilitating vegetation succession after disturbance (Simões *et al.* 2009). Moreover, the *Cistus* genus is a good candidate to test within leaf cohort trait covariations. The 21 species belonging to this genus are in fact considered as seasonal dimorphic semideciduous shrubs displaying two well-defined leaf cohorts during a year: summer leaves (SL) and winter leaves (WL) (Aronne and De Micco 2001). Summer leaves and WL strongly differ in anatomical, morphological and physiological leaf traits (Aronne and De Micco 2001; Catoni *et al.* 2012). Moreover, Puglielli *et al.* (2017a) recently found that the relationship between LMA and photosynthetic rate per unit of leaf dry mass followed seasonal changes in three *Cistus* spp. from different provenances, thus increasing the rationale to test for leaf cohort trait covariation patterns at a broader scale.

Considering the different periods of the year in which *Cistus* spp. form their leaves, we hypothesize that SL are characterized by a low return strategy while WL by a higher return strategy as they develop after the first rains following summer drought (i.e. more favourable conditions). Thus, in the two leaf cohorts the relationships among functional leaf traits could vary between them and from that expected on the basis of the LES.

In order to verify our hypothesis, we first summarized the differences in leaf traits and their pattern of covariation between SL and WL by collecting data from literature. Then, we compared the obtained patterns against those reported in Glopnet. In particular, we compared our results with both the global spectrum of evergreens, since in Glopnet *Cistus* spp. are classified as evergreen species, and global deciduous spectrum being *Cistus* spp. semideciduous species.

## Methods

### Construction of the database of morphological and physiological leaf traits of WL and SL

An extensive literature survey was carried out to identify the published studies on *Cistus* spp. The search terms for the three Scopus and Web of Science queries were: (i) ((‘*Cistus*’) AND (‘photosynthesis’ OR ‘photosynthetic rate’)), (ii) ((‘*Cistus*’) AND (‘leaf structure’ OR ‘leaf morphology’ OR ‘specific leaf area’ OR ‘leaf mass per area’ OR ‘SLA’ OR ‘LMA’)) and (iii) ((‘*Cistus*’) AND (‘nitrogen’)). Altogether, 142 studies covering the years 1987–2017 were identified.

The following leaf traits were included: LMA and its underlying components such as LTD (mg cm^−3^) and LT (μm). Leaf thickness values obtained from direct anatomical measurements, which are considered to be the most reliable estimate (see Niinemets 2015 for further discussion), were retained. When LTD was not available in the data source, it was derived as LMA/LT (Puglielli *et al.* 2015). The rationale to include LT and LTD in our database is that they can alter photosynthesis in reverse directions in woody plants, acting as a potential confounding effect in interpreting the bivariate relationships between LMA and photosynthesis (Niinemets 1999). Among the physiological leaf traits, we included net photosynthetic rate per unit of leaf area (A_a_, μmol CO_2_ m^−2^ s^−1^) and per unit of dry mass (A_m_, nmol CO_2_ g^−1^ s^−1^). The biochemical traits included leaf nitrogen content per unit dry mass (N_m_, %) and area (N_a_, g m^−2^). Since most of the studies generally reported photosynthesis and biochemical traits on an area basis, the traits on a mass basis were derived whenever LMA was available following Wright *et al.* (2004).

Overall, in order to include photosynthetic, biochemical and morphological leaf traits for both the cohorts in our database, we followed the standardized procedure developed by Niinemets (2015) with some modifications. In particular, the following three criteria were used to select the parameters to include in the database:

(i) Photosynthetic, biochemical and morphological leaf traits had to be sampled in the period November–December for WL (Puglielli *et al.* 2017a) and May–June for SL (Bongers *et al.* 2017). In general, we included in the database the maximum photosynthetic rate per each of the considered leaf cohorts. In fact, according to Niinemets (2015), we included the photosynthesis values measured under no stressful conditions because stress factors reduce stomatal conductance and photosynthetic rate also decreasing the leaf biochemical photosynthesis potentials. This could affect the results of the bivariate relationships between leaf morphological and physiological traits (Niinemets 2015). When possible, we analysed the reported seasonal trend in order to select the maximum photosynthetic rate per each of the considered leaf cohorts. If no seasonal trend was reported in a study, we included the photosynthetic rate whether it was sampled during the above-mentioned months range as reported in the Materials and Methods sections of our data sources and according to our expertise in the field. In addition, to be sure that plants were not in water stress conditions (if not specified as in Bongers *et al.* 2017), we checked stomatal conductance data for WL and SL. As such, we included in our database physiological data for stomatal conductance ≥ 150 mmol m^−2^ s^−1^ since above this threshold there is not an effect of water stress on photosynthesis according to Flexas *et al.* (2004). In particular, stomatal conductance range was 61–490 for SL and 132–580 for WL. The values below the selected threshold come from studies that explicitly reported the absence of water stress, as above mentioned.

(ii) Leaf traits had to be sampled in the field and (iii) on young fully developed apical leaves of adult plant. As adult plants we selected from the considered literature only 3 years old or more, since this threshold characterizes the reproductive individuals. We restricted the data acquisition to adult plant because differences in plant age can affect the estimates of the considered leaf functional traits (Niinemets 2015).

From the original studies, we also took geographical variables such as latitude (Lat, °), longitude (Long, °) and altitude (Alt, m a.s.l.). Concerning climatic variables, we had to follow a different approach to that generally employed to understand the climatic effect in shaping leaf traits (e.g. Wright *et al.* 2004; He *et al.* 2006; Niinemets 2015). Among our aims, we also wanted to identify the possible climatic drivers of leaf trait variations within single leaf cohorts. Considering that the selected leaf cohorts develop during a single growing season, the use of mean annual values of climatic variables as predictors would be meaningless. The following rationale was therefore used. Considering that *Cistus* spp. leaves take on average 20 days to fully develop under favourable environmental conditions (Crescente 1998), as that sampled here, we included in the database only mean monthly temperature (Temp, °C) and precipitation (P, mm) values relative to the previous month in which the leaves were sampled in our data sources.

Following data inclusion, we checked for eventual outliers. Firstly, we calculated traits mean and standard deviation (SD) within each leaf cohort; then, all the values falling outside the range mean ± 2 SD were considered outliers and removed from the database.

**Figure 1.**
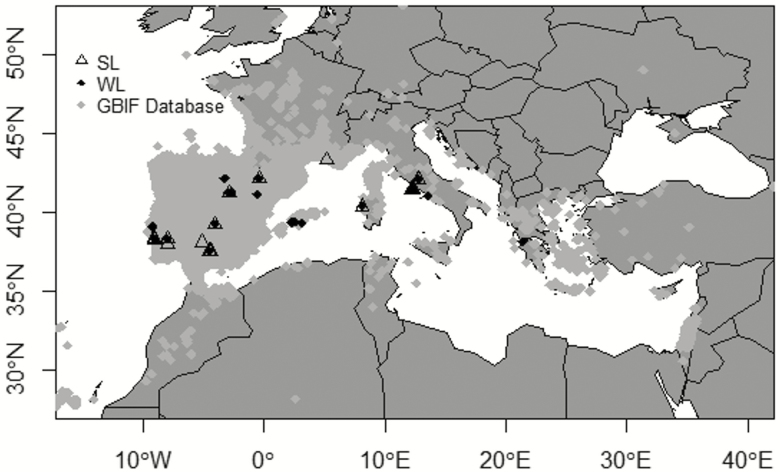
Geographical distribution of the sample locations for the considered leaf cohorts of *Cistus* species (SL = summer leaves, WL = winter leaves). Light grey rhomboidal symbols represent *Cistus* spp. presence points across the Mediterranean Basin according to the Global Biodiversity Information Facility (GBIF, http://www.gbif.org/species/2874026). Details on species and leaf traits sampled per each location are shown in **Supporting Information—Table 1**.

The applied procedure of study inclusion resulted in the final database comprising 38 studies (comprising three unpublished data sets from our laboratory) **[see****Supporting Information—Table S1****]** covering a significant portion of the Mediterranean Basin with observations from Portugal, Spain, France, Italy and Greece (Fig. 1). A summary of the bioclimatic variables for WL and SL is given in **Supporting Information—Table S2**. In particular, the database included data for nine *Cistus* spp., representing the 43 % of the species (i.e. 21 species in total, Correia and Ascensão 2017) belonging to this genus. At any rate, we were not able to obtain a database with all the values for the considered traits for WL and SL coming from the same data sources. This partially constrains the possibility to explicitly analyse the correspondence between the inherent variability of functional traits in SL and WL and their bivariate relationships.

### Data analysis

Pearson pairwise correlation coefficients were used to test for linear correlation among the considered leaf traits **[see****Supporting Information—Table S3****]**.

Standardized major axis (SMA) regression (Warton *et al.* 2006) was used to analyse relationships between LMA and its components (LTD and LT) against all the considered physiological and biochemical leaf traits as well as the relationship between LTD and LT. In all the analyses *Leaf Cohort* was used as the main factor. Differences in terms of slopes and intercepts were tested with the likelihood ratio and Wald statistic, respectively (see Warton *et al.* 2006 for further details). When pertinent, shifts between leaf cohorts along the common fitted slope were tested using the Wald statistic.

By using the function *slope.test* (smatr package version 3, Warton *et al.* 2012), SMA also allowed to test for significant differences between the obtained slopes for the bivariate relationships per each leaf cohort against that of the broad spectrum of evergreens and deciduous obtained from the Glopnet Database of Wright *et al.* (2004).

Standardized major axis regression was also carried out to analyse the relationships between the considered leaf traits and climatic variables (i.e. temperature and precipitation). All data (except the climatic data) were log-transformed for analyses that were run with the R library SMATR (Warton *et al.* 2012).

## Results

### Leaf trait variation in WL and SL

Through the entire database physiological traits had the highest variation while morphological traits showed the lowest one. Between the leaf cohorts, overall SL showed a lower variation than WL (Table 1). A_a_ was the most varying trait in WL (7.0-fold) while A_m_ had the highest variation in SL (6.1-fold). N_m_ and LT showed the narrowest range of variation in WL while N_a_ and LT in SL.

On average, WL showed 28 % and 22 % lower A_a_ and A_m_ than SL. At a biochemical level, no differences were found in N_a_ with an average value of 2.2 and 2.3 g m^−2^ in SL and WL, respectively, while N_m_ was 5 % higher in SL than in WL. Among morphological traits, LTD, LT and LMA were 54 %, 12 % and 2 % higher in SL compared to WL (Table 1).

**Table 1.** Means, minimum and maximum values (in parenthesis) for the physiological, biochemical and morphological leaf traits included in the analysis per each considered leaf cohort of *Cistus* species. SL = summer leaves; WL = winter leaves; A_a_ = net photosynthetic rate per unit of leaf area; A_m_ = net photosynthetic rate per unit per unit of leaf dry mass; N_a_ = nitrogen content per unit of leaf area; N_m_ = nitrogen content per unit of leaf dry mass; LMA = leaf mass area; LTD = leaf tissue density; LT = leaf thickness. Unit as well sample size per each trait is also shown.

Leaf traits	Unit	*n*	WL	*n*	SL
Physiological traits					
A_a_	μmol CO_2_ m^−2^ s^−1^	65	12.5 (2.9–23.0)	38	17.4 (5.8–25.0)
A_m_	nmol CO_2_ g^−1^ s^−1^	26	117.9 (33.4–242.1)	29	151.2 (37.4–266.4)
Biochemical traits					
N_a_	g m^−2^	11	2.3 (1.2–3.6)	15	2.2 (1.5–3.8)
N_m_	%	18	16.4 (13.4–22.4)	18	17.3 (10.0–27.0)
Morphological traits					
LMA	g m^−2^	40	130 (51–263)	44	132 (56–250)
LT	μm	17	179 (123–226)	26	200 (130–293)
LTD	mg cm^−3^	22	427 (160–816)	26	658 (372–1189)

### Relationship among morphological, physiological and biochemical leaf traits

**Figure 2.**
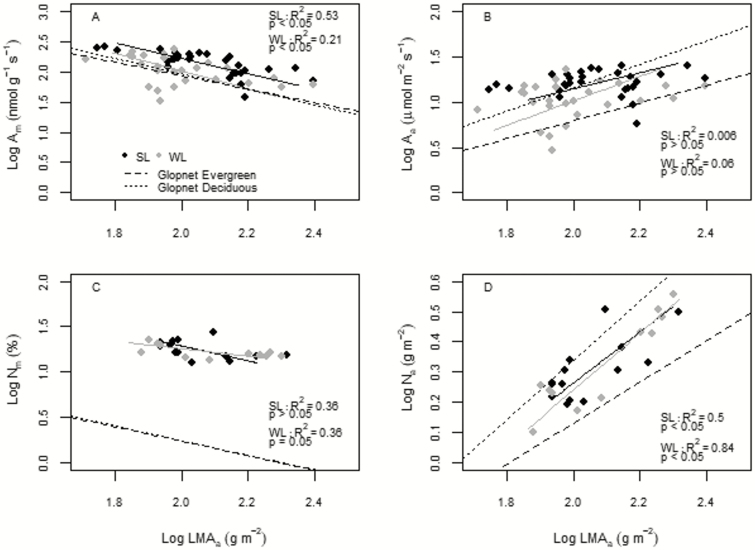
Log–log relationships between leaf mass area (LMA) with: (A) net photosynthetic rate per unit of leaf dry mass (A_m_) and (B) per unit of leaf area (A_a_), (C) nitrogen content per unit of leaf dry mass (N_m_) and (D) per unit of leaf area (N_a_) per each considered leaf cohort of *Cistus* species (SL = summer leaves, WL = winter leaves). *R*^2^ and *P*-value per each relationship are shown. The estimated slopes, intercepts, as well as the significance tests of the fitted slopes against that of the deciduous and evergreens spectrum from the Glopnet database (Wright *et al.* 2004) are given in Table 2. Relationships were considered significant at *P* < 0.05.

In general, the considered bivariate relationships on pooled data were in agreement with Glopnet (Table 2, Fig. 2). In fact, LMA scaled positively with N_a_ (*R*^2^ = 0.70, *P* < 0.0001) and negatively with A_m_ (*R*^2^ = 0.24, *P* = 0.0001) and *N*_m_ (*R*^2^ = 0.26, *P* = 0.009). Moreover, no significant relationship was found between LMA and A_a_ (*R*^2^ = 0.06, *P* = 0.07).

**Table 2.** Log–log relationships between leaf mass area (LMA) and: net photosynthetic rate per unit of leaf area (A_a_) and per unit of leaf dry mass (A_m_), nitrogen content per unit of leaf area (N_a_) and per unit of leaf dry mass (N_m_), leaf tissue density (LTD) and leaf thickness (LT) per each leaf cohort (WL = winter leaves, SL = summer leaves) of *Cistus* species as well as on pooled data (those for LTD–LMA and LTD–LT are not included since they were affected by *C. creticus* subsp. *eriocephalus* sample size, LT–LMA was not affected but it was removed as well). The relationship between LTD and LT is also shown. Slope, intercept and shift tests between the two leaf cohorts are shown. * indicates when the fitted slopes per each cohort were significantly different from that of the spectrum of deciduous (Dec) and evergreens (Ev) from the Glopnet database (Wright *et al.* 2004). NA = not available in Glopnet.

Relationship	Leaf cohort	*n*	Slope	Intercept	*R* ^2^	*P*	Shift.test	Dec	Ev
A_a_–LMA	WL	26	1.35a	−1.68a	0.06	0.231	0.0004	n.s.	n.s.
	SL	29	0.89a	−0.63a	0.006	0.696		n.s.	n.s.
	Pooled	55	1.26	−1.46	0.06	0.07		n.s.	n.s.
A_m_–LMA	WL	26	−1.47a	4.96a	0.21	0.019	–	n.s.	n.s.
	SL	29	−1.29a	4.80b	0.53	8.53E-05		n.s.	n.s
	Pooled	55	−1.41	4.94	0.24	0.0001		n.s.	n.s.
N_a_–LMA	WL	11	0.94a	−1.64	0.84	6.21E-05	0.559	*	*
	SL	14	0.84b	−1.43	0.50	0.0048		n.s.	n.s.
	Pooled	25	0.89	−1.53	0.70	1.74E-07		n.s.	*
N_m_–LMA	WL	11	−0.41a	2.08	0.36	0.05	0.364	*	*
	SL	14	−0.82b	2.93	0.23	0.087		n.s.	n.s.
	Pooled	25	−0.61	2.51	0.26	0.009		n.s.	n.s.
LTD–LMA	WL	22	1.93a	−1.09a	0.49	0.0002	–	NA	NA
	SL	26	0.81a	−0.19b	0.46	0.0001		NA	NA
LT–LMA	WL	15	−1.55a	5.29a	0.03	0.557	–	NA	NA
	SL	26	0.92a	0.36b	0.03	0.388		NA	NA
LTD–LT	WL	15	−0.80a	4.41a	0.71	8.40E-05	0.523	NA	NA
	SL	26	−0.75a	4.38a	0.36	0.001		NA	NA

These general patterns were confirmed both in WL and SL even if some differences were highlighted. A_m_ scaled negatively and N_a_ positively with LMA in both leaf cohorts (Table 2, Fig. 2). However, N_m_–LMA was not significant within each leaf cohort. Although no significant differences in slopes were found, intercepts significantly differed for A_m_ while a significant shift along the common axis was found for A_a_ (Table 2, Fig. 2). When the fitted slopes were tested against both the evergreens and deciduous from the Glopnet database, significant differences were found in WL for N_m_–LMA and N_a_–LMA (Table 2, Fig. 2).

**Figure 3.**
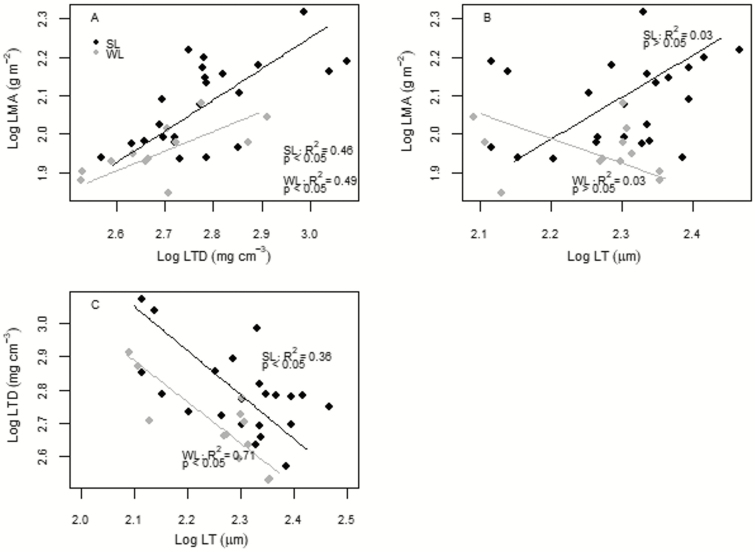
Log–log relationships between: (A) leaf tissue density (LTD) and leaf mass area (LMA), (B) leaf thickness (LT) and LMA and (C) LTD and LT per each considered leaf cohort of *Cistus* species (SL = summer leaves, WL = winter leaves). *R*^2^ and *P*-value per each relationship are shown. The estimated slopes and intercepts are given in Table 3. Relationships were considered significant at *P* < 0.05.

The covariation of LMA with its components revealed a significant relationship only for LTD–LMA in both the leaf cohorts (Table 2, Fig. 3). Moreover, LT and LTD were negatively correlated in both WL and SL (Table 2, Fig. 3). In the latter, LTD scaled negatively (*P* < 0.05) and LT positively (*P* < 0.05) with A_a_ and A_m_ (Table 3). Also, a positive relationship was found between N_a_ and LTD (Table 3). All the relationships between LTD and LT with the considered leaf physiological and biochemical parameters were not significant in WL (Table 3).

**Table 3.** Log–log relationships between (a) leaf tissue density (LTD) and (b) leaf thickness (LT) with: net photosynthetic rate per unit of leaf area (A_a_) and per unit of leaf dry mass (A_m_), nitrogen content per unit of leaf area (N_a_) and per unit of leaf dry mass (N_m_), per each leaf cohort (WL = winter leaves, SL = summer leaves) of *Cistus* species. Slope and intercept tests between the two leaf cohorts are shown.

	Relationship	Leaf cohort	*n*	Slope	Intercept	*R* ^2^	*P*
(a)	A_a_–LTD	WL	22	−1.17a	4.06	0.01	0.63
		SL	18	−1.01b	4.02	0.35	0.009
	A_m_–LTD	WL	22	−1.19a	5.12a	0.005	0.746
		SL	18	−1.38a	5.97b	0.67	3.55E-05
	N_a_–LTD	WL	5	0.79a	−1.87a	0.01	0.85
		SL	10	0.74a	−1.76a	0.40	0.047
	N_m_–LTD	WL	5	−0.95a	3.76a	0.25	0.39
		SL	10	−0.57a	2.83a	0.22	0.17
(b)	A_a_–LT	WL	15	−2.74a	7.15a	5E-04	0.936
		SL	18	1.74a	−2.78b	0.38	0.006
	A_m_–LT	WL	15	2.65a	−3.96a	3E-04	0.95
		SL	18	2.38a	−3.34a	0.4	0.005
	N_a_–LT	WL	5	−1.79a	4.34a	0.14	0.54
		SL	10	0.97a	−1.92b	0.05	0.52
	N_m_–LT	WL	5	2.15a	−3.72a	0.002	0.94
		SL	10	−0.74a	2.95b	0.11	0.34

### Dependency of leaf traits on climatic variables in WL and SL

The results of the trait–climate relationships highlighted no coordination between the selected traits and climatic variables in SL (Table 4). On the contrary, the generated models were mostly significant for WL. Within this leaf cohort, A_a_ and A_m_ increased, while N_m_ and LT decreased with temperature. On the other hand, N_a_ and LT increased with precipitation in WL while LTD decreased. Any relationship was found for LMA with both temperature and precipitation within each leaf cohort (Table 4).

**Table 4.** Bivariate relationships between temperature and precipitation against: net photosynthetic rate per unit of leaf area (A_a_) and per unit of leaf dry mass (A_m_), nitrogen content per unit of leaf area (N_a_) and per unit of leaf dry mass (N_m_), leaf mass area (LMA), leaf thickness (LT) and leaf tissue density (LTD) per each leaf cohort (WL = winter leaves, SL = summer leaves) of *Cistus* species. Sample size, slope and *R*^2^ are also shown. Different lowercase letters indicate significant differences between slopes at *P*-value ≤ 0.05 . Bold *R*^2^ indicates significant relationships at *P*-value < 0.05.

	Temperature				Precipitation			
	Leaf cohort	*n*	Slope	*R* ^2^	Leaf cohort	*n*	Slope	*R* ^2^
A_a_	WL	34	0.048a	**0.07**	WL	37	−0.0029a	0.08
	SL	34	0.0452a	0.02	SL	27	0.0012b	0.01
A_m_	WL	25	0.053a	**0.31**	WL	26	−0.0034a	0.12
	SL	27	0.079b	0.02	SL	25	0.0016b	0.00
N_a_	WL	6	0.031a	0.00	WL	6	0.0026a	**0.96**
	SL	9	−0.02a	0.36	SL	6	−0.002a	0.32
N_m_	WL	10	−0.0129a	**0.53**	WL	7	−0.0006a	0.00
	SL	12	−0.029b	0.01	SL	9	0.0032b	0.14
LMA	WL	33	0.037a	0.05	WL	26	−0.0034a	0.03
	SL	31	−0.0536a	0.03	SL	31	−0.0014b	0.00
LT	WL	15	−0.022a	**0.19**	WL	12	0.0029a	**0.30**
	SL	17	−0.0307a	0.01	SL	14	−0.0018a	0.00
LTD	WL	18	−0.0417a	0.11	WL	15	0.0029a	**0.21**
	SL	17	−0.053a	0.00	SL	21	0.0056b	0.00

## Discussion

### Leaf trait variations in WL and SL

In general, we found a great inherent variability in leaf traits in both WL and SL. LMA variation (from 51 to 263 g m^−2^ in WL and from 56 to 250 g m^−2^ in SL) fell in the range reported for higher plants in Poorter *et al.* (2009). A_a_ and A_m_ were highly variable in both the leaf cohorts (from 3.3- to 7.0-fold). In particular, in SL A_m_ is more variable (6.1-fold) than A_a_ (3.3-fold) according to results of Niinemets (1999), Wright *et al.* (2004), Kattge *et al.* (2011) while WL showed roughly the same magnitude of variation for both the parameters (6.3- and 7.0-fold for A_m_ and A_a_, respectively). This last result agrees with Niinemets (2015) who did not find a higher A_m_ variation than A_a_ for the Mediterranean *Quercus ilex* across its bioclimatic range of distribution. A greater variation in N_a_ (1.9-fold) than in N_m_ (0.7-fold) observed here for WL also agrees with the range of variation for these traits in previous extensive databases (e.g. Niinemets 1999; Wright *et al.* 2004; Kattge *et al.* 2011; Niinemets 2015). On the contrary, a similar variation in N_m_ (1.7-fold) and N_a_ (1.4-fold) was found in SL. At any rate, we observed a tendency of WL to have a higher N_a_ than SL, in line with previous findings (Werner and Máguas 2010; Correia and Ascensão 2017). Since WL tend to maximize resource acquisition in a short time because of their lower leaf lifespan (6 months), they invest more in leaf area than in dry mass. Thus, we argue that the greater N_a_ investment may be payed-back through the nutrient re-translocation during the inevitable leaf turnover occurring in spring, when the environmental conditions are favourable (see also Puglielli *et al.* 2017a). Considering that the nutrient re-translocation is a well-known process in *Cistus* genus (Milla *et al.* 2004; Dias *et al.* 2012; Simões *et al.* 2012; Correia and Ascensão 2017), this represents an additional strategy to minimize the necessity to invest in nutrient acquisition during the favourable period, thus maximizing growth and photosynthesis.

### Relationship among morphological, physiological and biochemical leaf traits

The considered bivariate relationships within each leaf cohort showed a relatively low explanatory power. A similar low explanatory power was found in studies involving congeneric species (see Muir *et al.* 2017 and references within). The modest coordination observed between leaf structure and physiology indicates that structural traits, such as LMA, are probably insufficient to identify the most important axes of trait variation in studies within genera. This suggests the possibility that there are many unique ways to vary leaf anatomy and photosynthesis without large effects on LMA (Tosens *et al.* 2012; Tomás *et al.* 2013; Muir *et al.* 2017). Ultimately, the obtained results reflect the ‘boundary line’ trade-offs proposed by Grubb (2015) who stated that such trade-offs set a soft constrain on evolutionary divergence making the correlations weaker than expected on the basis of the assumption of a ‘true trade-off’ (see Grubb 2015 and Muir *et al.* 2017 for further discussion).

At a leaf cohort level, LMA mostly scaled at the same rate with all the considered leaf traits in WL and SL except for the relationships N_a_-LMA and N_m_-LMA, and the latter was not significant within each leaf cohort. Nevertheless, at common LMA, SL showed a higher N_m_, LTD, LT and a lower A_m_, than WL. These results highlight that SL invest more resources in supportive structures (Niinemets 1999) necessary to face the environmental cues of the Mediterranean summers (Bongers *et al.* 2017). This view is supported by differences in LTD as a proxy of the leaf construction costs (de la Riva *et al.* 2016). Since SL have a longer leaf longevity (~10 months), a higher LTD can be the result of a greater foliar payback time (i.e. a longer leaf lifespan) as supported by the negative scaling of LTD with A_a_ and A_m_. Yet, unlike the lack of correlation generally found between LTD and LT (de la Riva *et al.* 2016), our results showed a negative correlation between them. Such negative correlation, associated to the absence of correlation between LT and LMA could reflect the capacity of *Cistus* spp. to modify LT to a greater extent in order to exert a positive morphological photosynthetic control at any given LMA. The LT control on the assimilation processes is evident in SL, as LT positively and significantly scales with both A_a_ and A_m_. Even if LT variation cannot require a long lifespan to pay back its construction costs (de la Riva *et al.* 2016), however, the degree of LT variation in SL seems to be constrained at relatively high LTD (i.e. a lower scatter of the points around the regression line), which in turn led to reduce photosynthesis. To compensate, SL showed a higher N_a_ with increasing LTD. This is not surprising, since leaves with a greater density generally need a higher N concentration to photosynthesize as leaves with lower density (Niinemets 1999). This strategy may drive the observed shift in A_a_ to higher values in SL.

On the other hand, in WL only the relationships A_m_–LMA, LTD–LMA and N_a_–LMA were significant, even if a lower degree of leaf structural control on photosynthesis was generally observed (i.e. a lower *R*^2^ for the relationship A_m_–LMA and no significant relationships LT–A_m_ and LTD–A_m_). The different WL pattern can be due to their shorter leaf lifespan meaning that WL do not need to mirror changes in leaf morphology with a longer payback time according to Puglielli *et al.* (2017a). Thus, as discussed in the previous subsection nitrogen economy of WL can be the driver of the differences in slope found for the relationship N_a_–LMA and N_m_–LMA between WL and SL and also between WL and the broad spectrum of evergreens and deciduous.

Interestingly, the two leaf cohorts converged in the lack of a structural control on N_m_ (even if the relationship on pooled data was significant), supporting that re-translocation may be the process involved in affecting nitrogen allocation patterns in *Cistus* spp. Niinemets (2015) found similar results for *Q. ilex* justifying it through the lack of a significant structural control on N_m_ that might identify water rather than N availability as the primary limitation in *Q. ilex* natural range. Our results support this view, and confirm that leaf cohorts can reshape the trade-offs between leaf functional traits as predicted by the LES.

### Dependency of leaf traits on climatic variables in WL and SL

Concerning the climatic drivers of leaf trait variations within single leaf cohorts, we found a lack of a significant explanatory power in SL while the contrary was observed in WL. Such results may be linked to a larger degree of variability in early winter bioclimatic characteristics of the sampled sites. Based on our results, it is therefore evident that changes in the early winter conditions through the Mediterranean Basin may represent a critical factor for WL structuring and functioning. On the other hand, the lack of dependency of SL leaf traits on bioclimatic variables may reflect a convergent evolution for this leaf cohort within *Cistus* genus, possibly due to a reduced degree of variability in summer under Mediterranean climatic conditions. Similarly, He *et al.* (2006) speculated a functional convergence of leaf trait relationships in an extreme environment such as Tibetan plateau from the lack of significance for the relationships between leaf traits and climate variables.

## Conclusions

Our results show the existence of a ‘within leaf cohort’ spectrum, which can diverge from that of evergreens and Deciduous. However, WL and SL differ among them since WL reflect a high-return strategy *sensu*Wright *et al.* (2004) while SL clearly display a low-return strategy. As such, the results contribute to widen the applicability of the LES framework shedding light on an important source of leaf morpho-physiological differentiation. This is particularly relevant considering that functional differences among leaf flushes formed at different times during a growing season are expected to increase due to global climate change (Niinemets 2014). Accordingly, these data could improve the ecological predictive models aimed to forecast species response to environmental changes.

## Sources of Funding

None.

## Contributions by the Authors

G.P. conceived the idea. G.P. and L.V. equally contributed to data gathering, database construction, statistical analysis and manuscript writing.

## Conflict of Interest

None declared.

## Supplementary Material

Supplementary Tables

## Supporting Information

The following additional information is available in the online version of this article—

**Table S1.** Geographic information of sample sites, leaf traits available and data sources per each leaf cohort of *Cistus* species.

**Table S2.** Bioclimatic variables relative to each leaf cohort of *Cistus* species.

**Table S3.** Pearson pairwise correlation coefficients for the considered physiological, biochemical and morphological leaf traits.
