# Supplementary material for: Inherent variation of functional traits in winter and summer leaves of Mediterranean seasonal dimorphic species: evidence of a ‘within leaf cohort’ spectrum
Source: AoB Plants. 2018 Apr 24;10(3):ply027. doi: 10.1093/aobpla/ply027 (PMC5965093; doi:10.1093/aobpla/ply027)
Supplement: Supplementary Tables [file ply027_suppl_supplementary_tables.docx]

**Table S1.** Geographic information of sample sites, leaf traits available and data sources per each leaf cohort (WL = winter leaves, SL = summer leaves) of *Cistus* species. A_a_ = net photosynthetic rate per unit of leaf area, A_m_ = net photosynthetic rate per unit per unit of leaf dry mass, N_a_ = nitrogen content per leaf area, N_m_ = nitrogen content per leaf dry mass, LMA = leaf dry mass per unit area, LTD = leaf tissue density, LT = total leaf thickness.

| Species | Latitude | Longitude | Altitude | Leaf Cohort | Traits available | Reference |
| --- | --- | --- | --- | --- | --- | --- |
|  |  |  |  |  |  |  |
| *C. creticus* subsp. *eriocephalus* | 41.03 | 13.55 | 9 | WL | A_a_, A_m_, LMA | Arena *et al.* (2013) |
| *C. creticus* subsp. *eriocephalus* | 41.03 | 13.55 | 9 | WL | LT | Aronne and De Micco (2001) |
| *C. creticus* subsp. *eriocephalus* | 41.03 | 13.55 | 9 | SL | LT | Aronne and De Micco (2001) |
| *C. creticus* subsp. *eriocephalus* | 41.45 | 12.26 | 1 | WL | A_a_, A_m_, LMA, LTD, LT | Bombelli and Gratani (2003) |
| *C. creticus* subsp. *eriocephalus* | 41.45 | 12.26 | 1 | SL | A_a_, A_m_, LMA, LTD, LT | Bombelli and Gratani (2003) |
| *C. albidus* | 37.55 | -4.51 |  | SL | A_a_, A_m_, LMA, LTD | Bongers et al. (2017) |
| *C. albidus* | 37.55 | -4.51 |  | WL | A_a_, A_m_, LMA, LTD | Bongers et al. (2017) |
| *C. albidus* | 37.55 | -4.51 |  | SL | A_a_, A_m_, LMA, LTD | Bongers et al. (2017) |
| *C. albidus* | 37.55 | -4.51 |  | WL | A_a_, A_m_, LMA, LTD | Bongers et al. (2017) |
| *C. salvifolius* | 37.55 | -4.51 |  | SL | A_a_, A_m_, LMA, LTD | Bongers et al. (2017) |
| *C. salvifolius* | 37.55 | -4.51 |  | WL | A_a_, A_m_, LMA, LTD | Bongers et al. (2017) |
| *C. salvifolius* | 37.55 | -4.51 |  | SL | A_a_, A_m_, LMA, LTD | Bongers et al. (2017) |
| *C. salvifolius* | 37.55 | -4.51 |  | WL | A_a_, A_m_, LMA, LTD | Bongers et al. (2017) |
| *C. ladanifer* | 37.55 | -4.51 |  | SL | A_a_, A_m_, LMA, LTD | Bongers et al. (2017) |
| *C. ladanifer* | 37.55 | -4.51 |  | WL | A_a_, A_m_, LMA, LTD | Bongers et al. (2017) |
| *C. ladanifer* | 37.55 | -4.51 |  | SL | A_a_, A_m_, LMA, LTD | Bongers et al. (2017) |
| *C. ladanifer* | 37.55 | -4.51 |  | WL | A_a_, A_m_, LMA, LTD | Bongers et al. (2017) |
| *C. populifolius* | 37.55 | -4.51 |  | SL | A_a_, A_m_, LMA, LTD | Bongers et al. (2017) |
| *C. populifolius* | 37.55 | -4.51 |  | WL | A_a_, A_m_, LMA, LTD | Bongers et al. (2017) |
| *C. salvifolius* | 41.53 | 12.28 | 53 | WL | A_a_, A_m_, LMA, LTD, LT | Catoni *et al.* (2012) |
| *C. creticus* subsp. *eriocephalus* | 41.53 | 12.28 | 53 | WL | A_a_, A_m_, LMA, LTD, LT | Catoni *et al.* (2012) |
| *C. monspeliensis* | 41.53 | 12.28 | 53 | WL | A_a_, A_m_, LMA, LTD, LT | Catoni *et al.* (2012) |
| *C. salvifolius* | 41.53 | 12.28 | 53 | SL | A_a_, A_m_, LMA, LTD, LT | Catoni *et al.* (2012) |
| *C. creticus* subsp. *eriocephalus* | 41.53 | 12.28 | 53 | SL | A_a_, A_m_, LMA, LTD, LT | Catoni *et al.* (2012) |
| *C. monspeliensis* | 41.53 | 12.28 | 53 | SL | A_a_, A_m_, LMA, LTD, LT | Catoni *et al.* (2012) |
| *C. ladanifer* | 38.27 | -9.01 | 132 | WL | A_a_, A_m_, LMA | Clemente *et al.* (2005) |
| *C. monspeliensis* | 38.27 | -9.01 | 132 | SL | A_a_, A_m_, LMA | Clemente *et al.* (2005) |
| *C. crispus* | 39.08 | -9.19 | 165 | WL | LMA | Correia *et al.* (2014) |
| *C. salvifolius* | 39.08 | -9.19 | 165 | WL | LMA | Correia *et al.* (2014) |
| *C. crispus* | 39.08 | -9.19 | 165 | WL | LMA | Correia *et al.* (2016) |
| *C. salvifolius* | 39.08 | -9.19 | 165 | WL | LMA | Correia *et al.* (2016) |
| *C. monspeliensis* | 40.37 | 8.1 | 1 | WL | A_a_, A_m_, LMA | de Dato *et al.* (2013) |
| *C. albidus* | 37.56 | -4.46 | 192 | SL | LMA, LTD, LT, N_a_, N_m_ | de la Riva *et al.* (2016) |
| *C. crispus* | 37.56 | -4.46 | 192 | SL | LMA, LTD, LT, N_a_, N_m_ | de la Riva *et al.* (2016) |
| *C. ladanifer* | 38.1 | -5.1 | 536 | SL | LMA, LTD, LT, N_a_, N_m_ | de la Riva *et al.* (2016) |
| *C. monspeliensis* | 38.1 | -5.1 | 536 | SL | LMA, LTD, LT, N_a_, N_m_ | de la Riva *et al.* (2016) |
| *C. ladanifer* | 38.29 | -9.01 | 130 | WL | N_m_ | Dias *et al.* (2012) |
| *C. monspeliensis* | 40.37 | 8.1 | 1 | WL | N_m_ | Gavrichkova et al. (2017) |
| *C. creticus* subsp. *eriocephalus* | 41.45 | 12.26 | 1 | SL | A_a_, A_m_, LMA, LTD, LT | Gratani and Bombelli (1999) |
| *C. creticus* subsp. *eriocephalus* | 41.45 | 12.26 | 14 | WL | A_a_ | Gratani and Varone (2004) |
| *C. creticus* subsp. *eriocephalus* | 41.45 | 12.26 | 14 | WL | A_a_ | Gratani and Varone (2004) |
| *C. creticus* subsp. *eriocephalus* | 41.45 | 12.28 |  | SL | A_a_ | Gratani and Varone (2004) |
| *C. creticus* subsp. *eriocephalus* | 41.45 | 12.28 |  | SL | A_a_ | Gratani and Varone (2004) |
| *C. creticus* subsp. *eriocephalus* | 41.45 | 12.28 |  | SL | A_a_, A_m_, LMA, LTD, LT | Gratani and Varone (2006) |
| *C. creticus* subsp. *eriocephalus* | 41.45 | 12.28 |  | SL | A_a_, A_m_, LMA, LTD, LT | Gratani and Varone (2006) |
| *C. creticus* subsp. *eriocephalus* | 41.45 | 12.28 |  | SL | A_a_, A_m_, LMA, LTD, LT | Gratani and Varone (2006) |
| *C. creticus* subsp. *eriocephalus* | 41.45 | 12.28 |  | SL | A_a_, A_m_, LMA, LTD, LT | Gratani and Varone (2006) |
| *C. creticus* subsp. *eriocephalus* | 41.45 | 12.28 |  | WL | A_a_, A_m_, LMA, LTD, LT | Gratani and Varone (2006) |
| *C. creticus* subsp. *eriocephalus* | 41.45 | 12.28 |  | SL | A_a_, A_m_, LMA, LTD, LT | Gratani and Varone (2006) |
| *C. creticus* subsp. *eriocephalus* | 41.45 | 12.28 |  | WL | A_a_, A_m_, LMA, LTD, LT | Gratani and Varone (2006) |
| *C. creticus* subsp. *eriocephalus* | 41.53 | 12.28 | 53 | WL | A_a_ | Gratani *et al.* (2008) |
| *C. creticus* subsp. *eriocephalus* | 41.53 | 12.28 | 53 | SL | A_a_ | Gratani *et al.* (2008) |
| *C. creticus* subsp. *eriocephalus* | 41.53 | 12.28 | 53 | WL | A_a_ | Gratani *et al.* (2008) |
| *C. creticus* subsp. *eriocephalus* | 41.53 | 12.28 | 53 | WL | A_a_, A_m_, LMA, LTD, LT, N_a_, N_m_ | Gratani *et al.* (upublished 2008, 2009, 2013) |
| *C. creticus* subsp. *eriocephalus* | 41.53 | 12.28 | 53 | SL | A_a_, A_m_, LMA, LTD, LT, N_a_, N_m_ | Gratani *et al.* (upublished 2008, 2009, 2013) |
| *C. creticus* subsp. *eriocephalus* | 41.53 | 12.28 | 53 | WL | A_a_, A_m_, LMA, LTD, LT, N_a_, N_m_ | Gratani *et al.* (upublished 2008, 2009, 2013) |
| *C. creticus* subsp. *eriocephalus* | 41.53 | 12.28 | 53 | WL | A_a_, A_m_, LMA, LTD, LT, N_a_, N_m_ | Gratani *et al.* (upublished 2008, 2009, 2013) |
| *C. creticus* subsp. *eriocephalus* | 41.53 | 12.28 | 53 | SL | A_a_, A_m_, LMA, LTD, LT, N_a_, N_m_ | Gratani *et al.* (upublished 2008, 2009, 2013) |
| *C. salvifolius* | 41.53 | 12.28 | 53 | WL | A_a_, A_m_, LMA, LTD, LT, N_a_, N_m_ | Gratani *et al.* (upublished 2008, 2009, 2013) |
| *C. salvifolius* | 41.53 | 12.28 | 53 | SL | A_a_, A_m_, LMA, LTD, LT, N_a_, N_m_ | Gratani *et al.* (upublished 2008, 2009, 2013) |
| *C. salvifolius* | 41.53 | 12.28 | 53 | WL | A_a_, A_m_, LMA, LTD, LT, N_a_, N_m_ | Gratani *et al.* (upublished 2008, 2009, 2013) |
| *C. salvifolius* | 41.53 | 12.28 | 53 | WL | A_a_, A_m_, LMA, LTD, LT, N_a_, N_m_ | Gratani *et al.* (upublished 2008, 2009, 2013) |
| *C. salvifolius* | 41.53 | 12.28 | 53 | SL | A_a_, A_m_, LMA, LTD, LT, N_a_, N_m_ | Gratani *et al.* (upublished 2008, 2009, 2013) |
| *C. monspeliensis* | 41.53 | 12.28 | 53 | WL | A_a_, A_m_, LMA, LTD, LT, N_a_, N_m_ | Gratani *et al.* (upublished 2008, 2009, 2013) |
| *C. monspeliensis* | 41.53 | 12.28 | 53 | SL | A_a_, A_m_, LMA, LTD, LT, N_a_, N_m_ | Gratani *et al.* (upublished 2008, 2009, 2013) |
| *C. monspeliensis* | 41.53 | 12.28 | 53 | WL | A_a_, A_m_, LMA, LTD, LT, N_a_, N_m_ | Gratani *et al.* (upublished 2008, 2009, 2013) |
| *C. monspeliensis* | 41.53 | 12.28 | 53 | WL | A_a_, A_m_, LMA, LTD, LT, N_a_, N_m_ | Gratani *et al.* (upublished 2008, 2009, 2013) |
| *C. monspeliensis* | 41.53 | 12.28 | 53 | SL | A_a_, A_m_, LMA, LTD, LT, N_a_, N_m_ | Gratani *et al.* (upublished 2008, 2009, 2013) |
| *C. creticus* subsp. *eriocephalus* | 41.41 | 12.22 | 14 | WL | A_a_, A_m_, LMA, LTD, LT, N_a_, N_m_ | Gratani *et al.* (upublished 2008, 2009, 2013) |
| *C. creticus* subsp. *eriocephalus* | 41.41 | 12.22 | 14 | SL | A_a_, A_m_, LMA, LTD, LT, N_a_, N_m_ | Gratani *et al.* (upublished 2008, 2009, 2013) |
| *C. salvifolius* | 41.42 | 12.23 | 14 | WL | A_a_, A_m_, LMA, LTD, LT, N_a_, N_m_ | Gratani *et al.* (upublished 2008, 2009, 2013) |
| *C. salvifolius* | 41.42 | 12.23 | 14 | SL | A_a_, A_m_, LMA, LTD, LT, N_a_, N_m_ | Gratani *et al.* (upublished 2008, 2009, 2013) |
| *C. creticus* subsp. *eriocephalus* | 42.08 | 12.79 | 350 | WL | A_a_, A_m_, LMA, LTD, LT, N_a_, N_m_ | Gratani *et al.* (upublished 2008, 2009, 2013) |
| *C. creticus* subsp. *eriocephalus* | 42.08 | 12.79 | 350 | SL | A_a_, A_m_, LMA, LTD, LT, N_a_, N_m_ | Gratani *et al.* (upublished 2008, 2009, 2013) |
| *C. creticus* subsp. *eriocephalus* | 42.08 | 12.8 | 550 | WL | A_a_, A_m_, LMA, LTD, LT, N_a_, N_m_ | Gratani *et al.* (upublished 2008, 2009, 2013) |
| *C. creticus* subsp. *eriocephalus* | 42.08 | 12.8 | 550 | SL | A_a_, A_m_, LMA, LTD, LT, N_a_, N_m_ | Gratani *et al.* (upublished 2008, 2009, 2013) |
| *C. creticus* subsp. *eriocephalus* | 42.09 | 12.82 | 750 | WL | A_a_, A_m_, LMA, LTD, LT, N_a_, N_m_ | Gratani *et al.* (upublished 2008, 2009, 2013) |
| *C. creticus* subsp. *eriocephalus* | 42.09 | 12.82 | 750 | SL | A_a_, A_m_, LMA, LTD, LT, N_a_, N_m_ | Gratani *et al.* (upublished 2008, 2009, 2013) |
| *C. monspeliensis* | 39.3 | 3.12 |  | WL | A_a_ | Gulias *et al.* (2009) |
| *C. monspeliensis* | 39.3 | 3.12 |  | WL | A_a_ | Gulias *et al.* (2009) |
| *C. monspeliensis* | 39.37 | 2.58 |  | WL | A_a_ | Gulias *et al.* (2009) |
| *C. monspeliensis* | 39.37 | 2.58 |  | WL | A_a_ | Gulias *et al.* (2009) |
| *C. monspeliensis* | 39.37 | 2.31 |  | WL | A_a_ | Gulias *et al.* (2009) |
| *C. monspeliensis* | 39.37 | 2.31 |  | WL | A_a_ | Gulias *et al.* (2009) |
| *C. monspeliensis* | 39.35 | 2.34 |  | WL | A_a_ | Gulias *et al.* (2009) |
| *C. monspeliensis* | 39.35 | 2.34 |  | WL | A_a_ | Gulias *et al.* (2009) |
| *C. albidus* | 39.3 | 3.12 |  | WL | A_a_ | Gulias *et al.* (2009) |
| *C. albidus* | 39.3 | 3.12 |  | WL | A_a_ | Gulias *et al.* (2009) |
| *C. albidus* | 39.37 | 2.58 |  | WL | A_a_ | Gulias *et al.* (2009) |
| *C. albidus* | 39.37 | 2.58 |  | WL | A_a_ | Gulias *et al.* (2009) |
| *C. albidus* | 39.37 | 2.31 |  | WL | A_a_ | Gulias *et al.* (2009) |
| *C. albidus* | 39.37 | 2.31 |  | WL | A_a_ | Gulias *et al.* (2009) |
| *C. salvifolius* | 39.37 | 2.31 |  | WL | A_a_ | Gulias *et al.* (2009) |
| *C. salvifolius* | 39.37 | 2.31 |  | WL | A_a_ | Gulias *et al.* (2009) |
| *C. salvifolius* | 38.38 | -9.11 |  | WL | A_a_ | Harley *et al.* (1987) |
| *C. salvifolius* | 38.38 | -9.11 |  | WL | A_a_ | Harley *et al.* (1987) |
| *C. salvifolius* | 38.38 | -9.11 |  | WL | A_a_ | Harley *et al.* (1987) |
| *C. salvifolius* | 38.38 | -9.11 |  | SL | A_a_ | Harley *et al.* (1987) |
| *C. salvifolius* | 38.38 | -9.11 |  | SL | A_a_ | Harley *et al.* (1987) |
| *C. clusii* | 41.23 | -2.9 |  | SL | LMA | Hernandez *et al.* (2011) |
| *C. clusii* | 41.23 | -2.9 |  | SL | LMA | Hernandez *et al.* (2011) |
| *C. clusii* | 41.23 | -2.9 |  | WL | LMA | Hernandez *et al.* (2011) |
| *C. clusii* | 41.23 | -2.9 |  | WL | LMA | Hernandez *et al.* (2011) |
| *C. clusii* | 41.23 | -2.9 |  | WL | LMA | Hernandez *et al.* (2011) |
| *C. creticus* subsp. *Creticus* | 38.14 | 21.44 | 250 | WL | N_m_ | Kytridis *et al.* (2008) |
| *C. creticus* subsp. *Creticus* | 38.14 | 21.44 | 250 | WL | N_m_ | Kytridis *et al.* (2008) |
| *C. creticus* subsp*. Creticus* | 38.14 | 21.44 | 250 | WL | N_m_ | Kytridis *et al.* (2008) |
| *C. albidus* | 43.29 | 5.18 |  | SL | N_m_ | Larcheveque et al. (2010) |
| *C. monspeliensis* | 40.37 | 8.1 | 144 | SL | A_a_, A_m_, LMA, N_a_, N_m_ | Liberati (2011) |
| *C. monspeliensis* | 40.37 | 8.1 | 144 | WL | A_a_, A_m_, LMA, N_a_, N_m_ | Liberati (2011) |
| *C. albidus* | 41.27 | -2.7 | 350 | WL | A_a_ | Llusià and Penuelas (2000) |
| *C. albidus* | 41.13 | -0.55 | 930 | WL | A_a_ | Llusià *et al.* (2010) |
| *C. albidus* | 41.13 | -0.55 | 930 | WL | A_a_ | Llusià *et al.* (2010) |
| *C. albidus* | 41.13 | -0.55 | 930 | WL | A_a_ | Llusià *et al.* (2010) |
| *C. albidus* | 41.13 | -0.55 | 930 | WL | A_a_ | Llusià *et al.* (2010) |
| *C. laurifolius* | 42.16 | -0.41 | 780 | SL | N_a_, N_m_ | Milla *et al.* (2007) |
| *C. laurifolius* | 42.16 | -0.41 | 780 | WL | LMA, N_a_, N_m_ | Milla *et al.* (2007) |
| *C. laurifolius* | 42.16 | -0.41 | 780 | SL | LMA, N_a_, N_m_ | Milla *et al.* (2007) |
| *C. laurifolius* | 42.16 | -0.41 | 780 | WL | LMA, N_a_, N_m_ | Milla *et al.* (2007) |
| *C. clusii* | 41.23 | -2.9 |  | WL | A_a_ | Munné-Bosch and Alegre (2002) |
| *C. clusii* | 41.23 | -2.9 |  | WL | A_a_ | Munné-Bosch and Alegre (2002) |
| *C. clusii* | 41.23 | -2.9 |  | WL | A_a_ | Munné-Bosch and Alegre (2002) |
| *C. monspeliensis* | 40.37 | 8.1 |  | SL | A_a_ | Nogués *et al.* (2015) |
| *C. creticus* subsp. *eriocephalus* | 42.08 | 12.79 | 350 | SL | A_a_ | Paolessi *et al.* (2015) |
| *C. creticus* subsp. *eriocephalus* | 42.08 | 12.8 | 550 | SL | A_a_ | Paolessi *et al.* (2015) |
| *C. creticus* subsp. *eriocephalus* | 42.09 | 12.82 | 750 | SL | A_a_ | Paolessi *et al.* (2015) |
| *C. ladanifer* | 39.25 | -4.04 |  | SL | A_a,_ A_m_, LMA | Parra and Moreno (2017) |
| *C. ladanifer* | 39.25 | -4.04 |  | WL | LMA | Parra and Moreno (2017) |
| *C. ladanifer* | 39.25 | -4.04 |  | WL | LMA | Parra and Moreno (2017) |
| *C. ladanifer* | 39.25 | -4.04 |  | SL | LMA | Parra and Moreno (2017) |
| *C. ladanifer* | 39.25 | -4.04 |  | SL | LMA | Parra and Moreno (2017) |
| *C. salvifolius* | 41.43 | 12.18 | 14 | SL | A_a_, A_m_, LMA, LTD, LT, N_a_, N_m_ | Puglielli *et al.* (2017 b) |
| *C. ladanifer* | 39.25 | -4.04 | 900 | SL | A_a_, A_m_, LMA | Ramirez *et al.* (2012) |
| *C. albidus* | 42.16 | -3.24 | 138 | WL | LMA, N_a_, N_m_ | Saura-Mas *et al.* (2010) |
| *C. monspeliensis* | 42.16 | -3.24 | 138 | WL | LMA, N_a_, N_m_ | Saura-Mas *et al.* (2010) |
| *C. salvifolius* | 42.16 | -3.24 | 138 | WL | LMA, N_a_, N_m_ | Saura-Mas *et al.* (2010) |
| *C. salvifolius* | 38.32 | -8.01 | 240 | SL | N_m_ | Simões et al. (2008) |
| *C. salvifolius* | 38.32 | -8.01 | 240 | WL | N_m_ | Simões et al. (2008) |
| *C. ladanifer* | 38.32 | -8.01 | 240 | SL | N_m_ | Simões et al. (2008) |
| *C. ladanifer* | 38.32 | -8.01 | 240 | WL | N_m_ | Simões et al. (2008) |
| *C. albidus* | 38 | -8 | 280 | SL | LMA, N_a_, N_m_ | Werner and Maguas (2010) |
| *C. monspeliensis* | 38 | -8 | 280 | SL | LMA, N_a_, N_m_ | Werner and Maguas (2010) |

**Table S2.** Means, minimum and maximum values (in parenthesis) for the considered bioclimatic variables relative to each leaf cohort. WL = winter leaves; SL = summer leaves.

|  | WL |  | SL |
| --- | --- | --- | --- |
| Latitude (°) | 40.23 (37.55-42.16) |  | 40.26 (37.55; 43.29) |
| Longitude (°) | 3.78 (-9.19-21.44) |  | 4.13(-9.11; 12.82) |
| Altitude (m a.s.l.) | 209 (1-930) |  | 268 (1 - 900) |
| Mean temperature (°C) | 15.96 (4.1-26.4) |  | 20.0 (13.7; 26.5) |
| Mean precipitation (mm) | 68 (0-250) |  | 69 (5; 622) |

**Table S3.** Pearson pairwise correlation coefficients for the considered physiological, biochemical and morphological leaf traits. *p* are shown in italic. Sample size is also shown. A_a_ = net photosynthetic rate per unit of leaf area, A_m_ = net photosynthetic rate per unit per unit of leaf dry mass, N_a_ = nitrogen content per leaf area, N_m_ = nitrogen content per leaf dry mass, LMA = leaf dry mass per unit area, LTD = leaf tissue density, LT = total leaf thickness.

|  | *n* | A_a_ | A_m_ | N_a_ | N_m_ | LMA | LT | LTD |
| --- | --- | --- | --- | --- | --- | --- | --- | --- |
| A_a_ | 88 |  | 0.77 | 0.11 | -0.14 | 0.24 | 0.40 | -0.10 |
|  |  |  | *0.0000* | *0.722* | *0.645* | *0.138* | *0.021* | *0.716* |
| A_m_ | 40 |  |  | -0.15 | 0.10 | -0.39 | 0.38 | -0.38 |
|  |  |  |  | *0.615* | *0.797* | *0.014* | *0.029* | *0.027* |
| N_a_ | 13 |  |  |  | -0.10 | 0.85 | 0.05 | 0.75 |
|  |  |  |  |  | *0.775* | *0* | *0.848* | *0.0012* |
| N_m_ | 13 |  |  |  |  | -0.48 | -0.23 | -0.38 |
|  |  |  |  |  |  | *0.015* | *0.413* | *0.158* |
| LMA | 40 |  |  |  |  |  | 0.23 | 0.68 |
|  |  |  |  |  |  |  | *0.154* | *0* |
| LT | 33 |  |  |  |  |  |  | -0.53 |
|  |  |  |  |  |  |  |  | *0.0004* |
| LTD | 33 |  |  |  |  |  |  |  |
